# Exposure to carbamate fungicide iodocarb does not affect reproductive behavior or milt volumes in precocious male brown trout (*Salmo trutta* L.) parr

**DOI:** 10.1007/s10695-020-00803-x

**Published:** 2020-04-15

**Authors:** K. Håkan Olsén, Hanna L. Olsén

**Affiliations:** School of Natural Science, Technology and Environmental Studies, Södertörn University, SE-141 89 Huddinge, Sweden

**Keywords:** Iodocarb, Milt volumes, Olfaction, Reproductive behavior, *Salmo trutta*

## Abstract

Previous studies with olfactory-disturbing pesticides resulted after exposure in disturbed behavior and physiology in fish. In the present experiment, reproductive behavior and milt volumes of precocious brown trout (*Salmo trutta* L.) male parr were studied in a large stream aquarium after exposure to the olfactory-disturbing fungicide 15 μg l^−1^ IPBC (iodocarb; 3-iodo-2-propynyl butyl carbamate) for 96 h. The statistical analyses did not reveal any significant differences for time attending females between controls and IPBC-exposed males. Furthermore, there were no significant differences in milt volumes. However, when taking all fish into consideration, there were significant differences in milt volumes between parr that had been attending females and those had not been attending females. Controls that had attended females had significantly higher milt volumes than controls or IPBC-exposed males that had not attended females. Taking all control and IPBC parr into consideration, there was a statistically significant positive correlation between time attended females and volume of milt and gonadosomatic index (GSI), respectively. In summary, 15 μg l^−1^ IPBC did not have any significant effects on mature male parr reproductive behavior and milt volumes.

## Introduction

Various types of pesticides are present in surface water, and there are concerns about their effects on aquatic organisms (e.g., Fulton et al. 2014; Hessel et al. 1997; Solomon et al. 2014). Studies have shown negative effects of pesticides on the chemical detection of molecules with an important role in fish and crustaceans (Olsén 2011, 2015). There are molecules used in communication as pheromones and others during foraging. Electro-physiological studies (electro-olfactogram, EOG) have shown that some pesticides reduce the olfactory response to the amino acids l-serine and l-histidine in juvenile coho salmon (*Oncorhynchus kisutch*) and rainbow trout (*O. mykiss*) (Jarrard et al. 2004; Tierney et al. 2006a, 2007). The behavioral responses to l-histidine were also affected. The carbamate fungicide IPBC (iodocarb; 3-iodo-2-propynyl butyl carbamate) was the most potent of the 10 compounds tested. It is used in antisapstain formulations in the forestry industry and in treatment of fresh lumber and during water runoff and can result in IPBC contamination of aquatic environments (Juergensen et al. 2000). Carbamates are *N*-methyl carbamates derived from carbamic acid (NH_2_COOH), and IPBC is one of the compounds. IPBC reduced EOG responses and preferences for l-histidine at concentrations as low as 1 μg l^−1^, about 100 times lower than the 96-h LC_50_ value for the most sensitive stage in juvenile coho salmon and rainbow trout (Bailey et al. 1999; Farrell et al. 1998). No observed effect concentration (NOEC) for juvenile coho salmon was set to < 0.70 mg l^−1^ (Farrell et al. 1998). Behavioral and physiological alarm reactions to skin extracts were also affected by ≥ 1 μg l^−1^ IPBC (Tierney et al. 2006b). The mechanisms behind the effects of IPBC are not fully understood but suggested to be connected to iodine (Canadian Council of Ministries of the Environment 1999). It has been shown to increase the acetylcholinesterase activity in the brain but not in the olfactory bulbs (Jarrard et al. 2004). IPBC is one of several preservatives in personal care products that can have negative effects on bacterial activity in sewage treatment plants and on their efficacy to degrade waste molecules (Carbajo et al. 2015). IPBC concentrations in sewage treatment plant effluents can be as high as or higher than in influent water, e.g., in one case, it was 65 ng l^−1^ in and 120 ng l^−1^ out (Tjus 2014). The toxicity of IPBC to fish and crustaceans varies between species. The lowest concentration with toxic effects (LOEC), 1.9 μg l^−1^, was observed during a 35-day exposure with fathead minnow (*Pimephales promelas*) (Juergensen et al. 2000). Another carbamate pesticide, carbofuran, significantly reduced the electrophysiological (EOG) response in mature male Atlantic salmon (*Salmo salar*) parr to the priming pheromone prostaglandin F_2α_ at a concentration as low as 1 μg l^−1^, and the threshold of detection was reduced 10-fold (Waring and Moore 1997). The same prostaglandin has been suggested as important parts of the priming female pheromone with effects in precocious brown trout male parr (Moore et al. 2002), but prostaglandins are not detected in rainbow trout (Laberge and Hara 2004). In brown trout, prostaglandin F_2α_ (PGF_2α_), prostaglandin F_1α_ (PGF_1α_), and the metabolite 15-keto prostaglandin F_2α_ are detected at low concentrations (Moore et al. 2002). Exposure to PGF_2α_ and PGF_1α_ (10^−8^ M) resulted in increased volumes of expressible milt and plasma concentrations of sex steroid hormones in precocious brown trout male parr. One of these hormones, 17,20β-dihydroxy-4-pregnen-3-one, is directly connected to the final maturation of sperms and their performance, and spermiation in different species of fish (e.g., Miura et al. 1992; Zheng et al. 1997; Scott et al. 2010; Hellström et al. 2012).

Previous studies with salmonid fish have shown that odors from ovulating females affect male behavior and physiology. Males are attracted to female-scented water (releaser effect) and contact with urine or ovarian fluid from ovulating females increases blood plasma sex hormone levels and volume of strippable milt (primer effect) (e.g., Olsén and Liley 1993). As mentioned before, prostaglandins are suggested to play an important part in primer effects in brown trout and Atlantic salmon (Moore and Waring 1996; Moore et al. 2002). Pesticides of various types have been shown to have negative effects on the olfactory sense and the detection of prostaglandins (Moore and Waring 1996). Studies have also shown that the very potent olfactory-disturbing insecticide cypermethrin (Moore and Waring 2001) affects reproductive behavior and endocrinology, including primer effects of ovarian fluid, in precocious brown trout (*Salmo trutta*) male parr (Jaensson et al. 2007). Similar effects were shown with copper sulfate (Jaensson and Olsén 2010) which is known to have negative effects on olfactory receptor cells (e.g., Bjerselius et al. 1993; Lari et al. 2019). In both these studies, precocious male brown trout parr courting behavior and proximity to digging females were affected. The aim of the present study was to investigate whether the olfactory-disturbing fungicide IPBC can affect mature male brown trout parr reproductive behavior and milt volumes. Groups of control and exposed parr were studied in an oval 35,000-l stream tank. Each parr was individually observed for ten 6-min periods, and the interactions with nest-digging females were recorded. After each experiment, all parr were collected, and the volume of expressible milt and the gonadosomatic index (GSI) were measured.

## Materials and methods

### Fish

Anadromous mature male and female brown trout (≥ 3.3-kg individual weight; Jaensson and Olsén 2010; Jaensson et al. 2007; Olsén et al. 1998) were captured in the autumn of 2008 during the spawning run in the Dalälven River (60° 34′ 2″ N; 17° 26′ 59″ E). The spawning period of the population is October–November. Adult fish are used in a breeding program to compensate for lack of natural reproduction caused by a hydroelectric dam that prevents upstream migration to the spawning grounds. We had access to a restricted number of adult males and females. They were kept at the National Board of Fisheries Research Station (60° 34′ 2″ N; 17° 26′ 59″ E). The females were checked once a week to detect ovulation. It was not possible to expose adult fish to IPBC, so instead, we used precocious males. The mature male parr were 2-year-old hatchery-reared fish sampled randomly from the station stock supply. Mature parr were sampled from large tanks at the hatchery. The precocious parr are darker in appearance than non-mature males. A low proportion of the 2-year-old parr was mature, 1–2% of the total number. There was no difference in weight between controls and IPBC-exposed mature parr (controls 126.0 ± 26.0, *N* = 15; IPBC 121.3 ± 25.0, *N* = 15; *P* > 0.05, *t* = 0.58, df = 28).

### Stock solution and concentrations

Stock solution was prepared by adding iodocarb (IPBC, 3-iodo-2-propynyl butyl carbamate; 97% pure; Sigma-Aldrich) to 95% ethanol to give a stock solution at a nominal concentration of 100 mg l^−1^ (measured concentration 91 mg l^−1^; chemical quantification was performed using liquid chromatography-mass spectrometry, LC-MS/MS, at the Department of Aquatic Science and Assessment, Section for Organic Environmental Chemistry and Ecotoxicology, Swedish University of Agricultural Sciences, SLU, Uppsala, Sweden; the detection limit was 0.5 μg l^−1^). The measured concentrations in the exposure tanks were 15.0 ± 1.0 μg l^−1^ (*n* = 3; mean ± SD; range 14–16 μg l^−1^; predicted concentration 15 μg l^−1^) directly after a water change (every 24 h during 96-h total exposure) and 5.8 ± 0.6 μg l^−1^ (*n* = 3; range 5.1–6.3 μg l^−1^) immediately prior to a water change. No IPBC was detected in the control water, which was water from the Dalälven River to which only a small amount of ethanol had been added. The chemical composition of Dalälven River water during exposures and groundwater used in the stream tank are given in Jaensson and Olsén (2010) (Table III). Groundwater was less turbid, making it easier to observe the fish and their behavior (Olsén et al. 1998; Jaensson et al. 2007; Jaensson and Olsén 2010).

### Exposure to IPBC

Parr were randomly selected from the stock tank with mature male parr and placed in clean, static, aerated, treatment tanks (total volume 400 l). The control group was treated with ethanol solvent (60 ml to 300-l river water). The treatment groups were exposed to 15 μg l^−1^ IPBC. The concentration should not be acutely toxic but about 10 times higher than the freshwater guideline value 1.9 μg l^−1^ (Canadian Council of Ministries of the Environment 1999). The guideline value is one-tenth of the 35-day LOEC for reduced weight gain and growth in embryos for fathead minnow (*Pimephales promelas*). An acute toxic test revealed that rainbow trout was the most sensitive of the six species tested, 96-h LC_50_ of 67 μg l^−1^ (Springborn Laboratories Inc. 1990, referred to in Canadian Council of Ministries of the Environment 1999). Water was changed every 24 h and treatment reapplied. Approximately 75% of the water was emptied before fresh river water was allowed to flow vigorously through the tank for at least 10 min. After this flushing period, the water was filled again to the assigned volume and turned off, and either ethanol or ethanol with IPBC was added (60 ml ethanol to 300 l). The water was continuously aerated, and the temperature varied between 7 and 10 °C. Throughout the exposure period, water samples were taken from exposure tanks and immediately frozen at − 20 °C for later analysis at SLU, Uppsala (see previous section). No mortality was observed during exposures or during behavior tests.

## Experiments

### Stream tank

The behavior experiments were performed during October 2008 in a stream tank (oval; 35,000 l) located in the Stream Water Ecology Laboratory at the Swedish National Board of Fisheries Research Station. Aerated groundwater was used to eliminate outside influences such as odors from other brown trout, and as the water was not turbid, recordings of fish behavior were easy. The water was continuously supplied, and the temperature was set at 8 °C. A turbine was located at the beginning of each long side to create a circular current. The bottom of each long side was covered with a thick layer of gravel. The photoperiod was set at a 12-h cycle (for further information about the stream tank, including a drawing and technical information, see Jaensson et al. (2007) and Olsén et al. (1998)).

### The experimental design

Before placing in the stream tank, parr were pre-exposed for 96 h to ethanol (60 ml to 300 l water) or 15 μg l^−1^ IPBC dissolved in ethanol (60 ml of the solution to 300 l water). On the third day of exposure, all parr were anesthetized and tagged with a numbered disc for individual recognition. The parr recuperated for 24 h in the same solution as before. About 12 h before starting the behavioral observations, two ovulating females were added to the stream tank. Two females were added to ensure that at least one started nest digging. When two or four adult males were added to the tank, males and females were placed on opposite sides of the tank. They were separated by nets. Early in the morning on day 4 of exposure, four parr from each treatment were added to the stream tank, and the nets were removed. After 1 h, behavior observations began. The experiments were repeated four times with new parr and adult fish. Adult males were used in two experiments, but as females also started nest digging with only parr present, adults were excluded in two experiments. The presence of adult males was not necessary to induce female nest digging.

### Behavior observations

Each fish was used only in one experiment. Each individual parr was observed for 6-min periods, ten times without predefined order. These were distributed as seven 6-min periods during day 1 (12 h) and three 6-min periods on the morning of day 2 (6 h) (Olsén et al. 1998). This gave 3600 s of observation time for each parr. After the final observation on day 1, the lights and turbines were turned off to prevent spawning at night. For each parr, the time attending a nest-digging female was recorded, and the time was summarized to one observation per fish. After the final behavior observation on day 2, the water level in the stream tank was reduced, and the fish were caught randomly, i.e., no pre-defined order, anesthetized, and the milt collected. Parr were stripped of their milt by gentle abdominal pressure, and the milt was delivered into pre-weighed glass tubes (Olsén and Liley 1993). Previous studies have shown that mature brown trout parr with intact olfactory sense are attending and staying in close proximity to digging females for a significantly longer time than anosmic males, and the former also have significantly higher volumes of strippable milt (Olsén et al. 1998). The control males are also courting females. Chemicals that affect the olfactory sense give the same effects as those seen in anosmic parr (Jaensson and Olsén 2010; Jaensson et al. 2007).

### Samples taken directly after experiments

After each experiment, the parr were anesthetized in 0.5% phenoxy ethanol, weighed, measured, and stripped of milt (Olsén and Liley 1993). The fish were killed by a blow on the head, followed by decapitation. The milt was weighed, and gonads removed and weighed to calculate the gonadosomatic index (GSI = ((gonad weight + stripped milt weight) / weight of fish) × 100).

### Statistical analyses

As we did not know the distribution of data, statistical comparisons were done with non-parametric tests. The Mann-Whitney test was used to compare two groups. The Kruskal-Wallis ANOVA test was used to compare more than two groups. Post hoc comparisons were performed using Dunn’s multiple comparisons test. Analysis of the correlation between time with females and milt volumes and GSI was performed using the Spearman rank correlation analysis. Parametric *t* test was used when comparing parr weights. The level of significance (two-tailed) was set at *P* = 0.05. Calculations and graphics were performed using the program GraphPad Prism™ (GraphPad Inc.: www.graphpad.com).

## Results

### Behavior

**Fig. 1:**
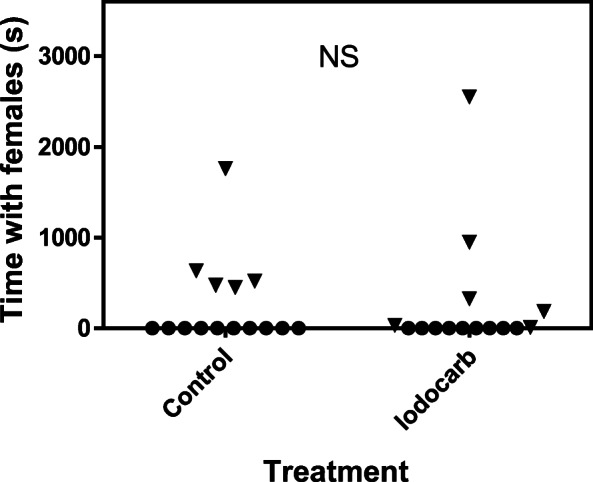
Time close to females in control parr and parr exposed to iodocarb (IPBC). Individuals marked with triangle were attending females. There were no differences between controls and exposed parr (Mann-Whitney test, *P* > 0.05) taking all fish in consideration or only males attending females

One spawning with an adult male was recorded; no parr was participating. There were no significant differences for the time attending females between the exposures (MW U = 111, *P* > 0.05; *N*_1_ = *N*_2_ = 15). Furthermore, there were no significant differences when taking only the parr attending females in consideration (MW U = 9; *P* > 0.05; *N*_1_ = 5, *N*_2_ = 6). Five of 15 control parr were recorded attending, courting, and spending some time close to a nest-digging female with or without adult males present (range 452–1762 s). Six of the 15 IPBC-exposed parr were attending and courting a female (range 11–2552 s) (Fig. 1). One parr from each treatment were excluded as they did not show any expressible milt and had not been attending a digging female. There was no significant difference in weight between parr that attended females and those that had not been attending females (both controls and exposed included 125.4 ± 9.1 g, *N* = 11; 122.6 ± 5.2 g, *N* = 19; *P* > 0.05, *t* = 0.29, df = 28).

### Milt volumes and GSI

**Fig. 2:**
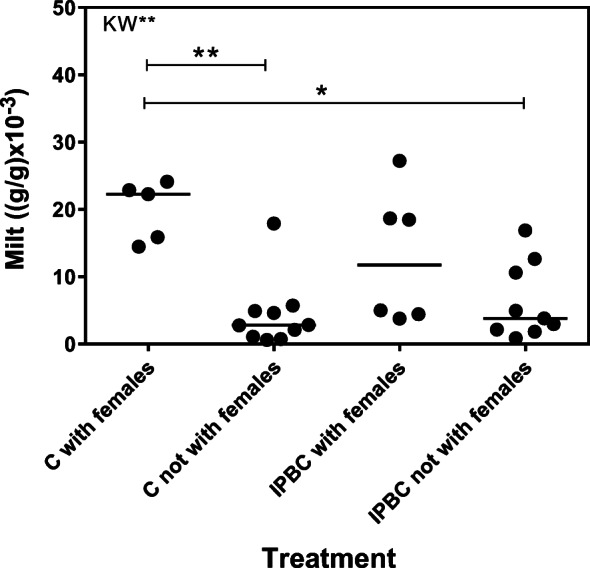
Volumes of expressible milt in control and IPBC-exposed mature male parr recorded attending digging females (“with females”) or not observed with females. Kruskal-Wallis ANOVA revealed significant differences between the four groups (KW** = *P* = 0.005). The ANOVA was followed by Dunn’s multiple comparisons test. Control parr attending digging females had significant higher volumes of milt compare with the two groups of parr not recorded to be with females (** = *P* < 0.01; * = *P* < 0.05)

**Fig. 3:**
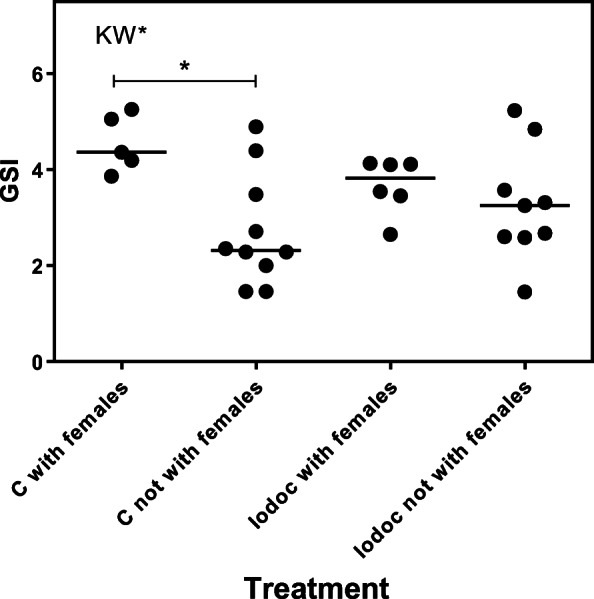
Gonadosomatic index (GSI) in control and IPBC-exposed mature male parr recorded attending digging females (“with females”) or not observed to attend females. Kruskal-Wallis ANOVA revealed significant differences between the four groups (KW* = *P* < 0.05). The following Dunn’s multiple comparisons test revealed a significant higher GSI in control attending females compare with controls not recorded to be with females (* = *P* < 0.05)

**Fig. 4:**
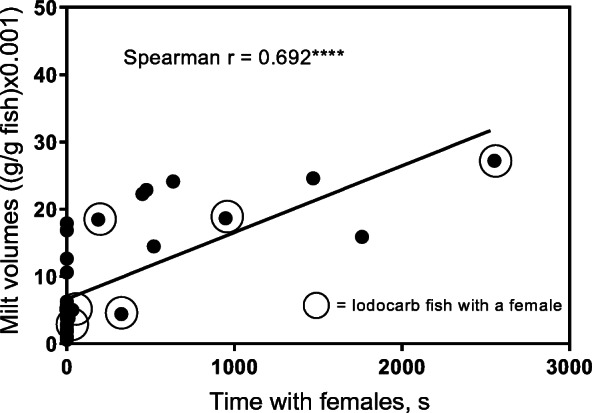
A positive correlation between total time attending digging females and milt volumes in mature parr (Spearman rank correlation analysis, *** = *P* < 0.001; number of pairs = 30). Each dot represents one individual. IPBC-exposed parr are surrounded by a circle

**Fig. 5:**
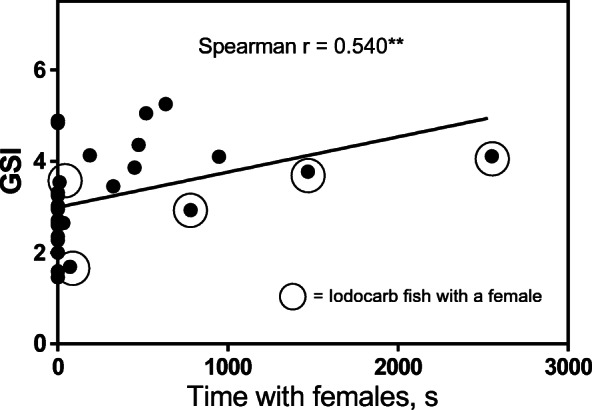
A positive correlation between total time attending digging females and GSI in mature parr (Spearman rank correlation analysis, ** = *P* < 0.01, number of pairs = 30). Each dot represents one individual. IPBC-exposed parr surrounded by a circle

There were no significant differences in milt volumes between control parr and parr exposed to IPBC (control: median 4.9 (g/g) × 10^−3^, min 0.6, max 24.1, *N* = 15; IPBC: median 4.9 (g/g) × 10^−3^, min 0.8, max 27.2, *N* = 15; MW U = 108, *P* > 0.05). Kruskal-Wallis ANOVA revealed that there were significant differences in milt volumes between parr that attended females and those that had not (Kruskal-Wallis statistics = 12.75; *P* = 0.005). Parr were divided into control groups and those that had been pre-exposed to IPBC (Fig. 2). The volumes of milt were significantly higher in control parr that had attended females (*N* = 5) compared with control parr (*N* = 10) or IPBC parr (*N* = 9) that had not been attending females (Dunn’s multiple comparisons test; mean rank difference 15.30, *P* < 0.01 and 12.62, *P* < 0.05, respectively). No significant differences were observed between control (*N* = 5) and IPBC-exposed males (*N* = 6) that had attended females (mean difference 5.07, *P* > 0.05). Kruskal-Wallis ANOVA showed statistical differences in GSI between the groups (Kruskal-Wallis statistics = 9.02; *P* = 0.03) (Fig. 3). Only controls attending females (*N* = 5) were significantly different from controls (*N* = 10) that had not been attending females (Dunn’s multiple comparisons test; mean rank difference 14.10, *P* < 0.05). Spearman’s rank correlation analysis showed that there was a significant positive correlation between total time with females and the volume of expressible milt (Spearman *r* = 0.692, *P* < 0.001, number of pairs = 30) (Fig. 4). There was also a significant positive correlation between total time with females and GSI (Spearman’s *r* = 0.540, *P* < 0.01, number of pairs = 30) (Fig. 5). IPBC-exposed parr interacting with females (marked by circles) were evenly distributed in time with females and milt volumes and time with females and GSI. This supports the idea that there were no differences between control males and IPBC-exposed males.

## Discussion

The fungicide IPBC did not have any significant effects on the interest of precocious male brown trout parr to stay with nest-digging ovulating females. There were no significant differences in time attended females between controls and parr exposed to IPBC when comparing all fish or only fish interacting with females. Previous studies with mature brown trout parr in the same behavior setup as the present have shown significant differences in courting behavior, time with females, and milt volumes after exposure to the potent olfactory-disturbing chemicals pyrethroid, insecticide cypermethrin, and copper sulfate (Jaensson and Olsén 2010; Jaensson et al. 2007). The pattern was the same as that observed with anosmic parr (Olsén et al. 1998). At low concentrations, these two chemicals have negative effects on olfactory senses in salmonid fish (e.g., Brown et al. 1982; Bjerselius et al. 1993; Moore and Waring 2001; Moran et al. 1991; Sandahl et al. 2004). The study by Moore and Waring (2001) is especially interesting as they used the proposed priming pheromone PGF_2α_ as an odor stimulus. IPBC, which was used in the present study, reduced the electrophysiological (EOG) and behavioral responses to the amino acid l-histidine in juvenile rainbow trout at concentrations as low as 1 μg l^−1^ (Tierney et al. 2007). The preference response to l-histidine was absent when EOG responses had decreased more than 60%. IPBC at 100 μg l^−1^ decreased the EOG response to l-serine to about 50% of the pre-exposure response, and after 60 min in clean water, the reduction was less, at about 25% (Tierney et al. 2006a). In an earlier study with coho salmon, Jarrard et al. (2004) found that the effective nominal IPBC concentration to reduce the EOG response to l-serine was as low as 1.28 μg l^−1^ and the highest concentration, 47.5 μg l^−1^, which reduced the response to about 20% of the 30 min pre-exposure. With the two other carbamates tested, carbofuran and mancozeb, the nominal concentrations to cause a 50% reduction in EOG amplitude was 10.4 μg l^−1^ and 2.05 mg l^−1^, respectively.

Tierney et al. (2006b) observed that 30-min IPBC exposure impaired behavior alarm responses to skin extract in juvenile coho salmon. Freezing behavior and dashing decreased with exposure to IPBC. Interestingly, swimming activity significantly increased in parr pre-exposed to 100 μg l^−1^ when skin extract was added. This may indicate that the fish could detect odors, or a certain type of odors, but that did not induce alarm reactions.

We do not know if IPBC affects the detection of priming prostaglandins in brown trout male parr, but a previous study with precocious male Atlantic salmon parr demonstrated that the insecticide carbofuran (2,3-dihydro-2,2-dimethyl-7-benzofuranyl-*N*-methylcarbamate), as low as 1 μg l^−1^, reduced the electrophysiological (EOG) response to PGF_2α_ and PGF_1α_ (Waring and Moore 1997). The carbamate also gave an 85% reduction of the EOG response to 10^−5^ M l-serine. Even more interesting, a 5-day exposure to 2.7 μg l^−1^ carbofuran significantly reduced the priming effect of the prostaglandins that is milt volumes and plasma levels of 17,20β-dihydroxy-4-pregnen-3-one. Precocious brown trout male parr detect the two prostaglandins at low concentrations, and exposures to water solutions give physiological priming effects as in salmon (Moore et al. 2002). The results demonstrate that carbofuran affects at least two different olfactory receptor types, l-serine and PGF_2α_ (Laberge and Hara 2004). The authors demonstrated that cross adaptation with l-serine did not change the EOG (olfactory epithelium) or EEG responses (olfactory bulb) to PGF_2α_ in brown trout. Low concentrations of carbofuran have also been shown to affect behavior responses to amino acids and food extracts in goldfish (*Carassius auratus*) (Saglio et al. 1996, 2003). Eight-hour exposure to 1 μg l^−1^ gave a significant reduction in attraction to extracts of chironomids (Saglio et al. 1996). Carbofuran is a specific insect cholinesterase inhibitor but is also toxic to vertebrates (Gupta 1994). IPBC that we studied in the present study has shown on the contrary to stimulate cholinesterase in the brain (Jarrard et al. 2004; the National Institute for Occupational Safety and Health, NIOSH).

The results of the tests with IPBC mentioned above are acute effects. In the present study, the fish were able to recover for at least 1 h before the first behavior recording, and the tests continued for 30 h. This may be one explanation for the differences between the present and previous studies. Recovery of olfactory functions may be high enough to induce a behavioral response even though EOG measurements still show a significant reduction in response (e.g., Razmara et al. 2019). Another explanation may be that different behaviors as well as different compounds and olfactory receptors were studied (e.g., Laberge and Hara 2004; Dew et al. 2014). Dew and collaborators observed that nickel and copper targeted different receptors and reduced the EOG response to l-alanine and taurocholic acid, respectively. In a study with channel catfish (*Ictalurus punctatus*), the authors found that the three morphologically distinct olfactory receptor cells, i.e., ciliated, microvillous, and crypt, use different transduction cascades, and there are distinct patterns of projects to the olfactory bulb (Hansen et al. 2003). Ciliated neurons project to regions of the olfactory bulbs responding to bile acids or amino acids and microvillous neurons responding to amino acids or nucleotide odorants. The olfactory ligands to cryptic neurons are not known, but they project to distinct areas of the olfactory bulbs (Hansen et al. 2003). Experiments with crucian carp (*Carassius carassius*) suggest that crypt cells express olfactory receptors for the pheromones prostaglandin F_2α_ and 17,20β-dihydroxy-4-pregnen-3-one (Hamdani and Døving 2006; Lastein et al. 2006). It has in salmonids been suggested that ciliated receptors are sensitive to bile acids and microvillous cells detect amino acids (Thommesen 1983). The results of other studies give support to those subsets of ciliated and microvillous olfactory neurons responding to amino acids (Hara and Zhang 1996; Hara and Zhang 1998). What kind of olfactory receptor cells and receptor molecules that are involved in the detection of prostaglandins acting as priming pheromones in brown trout and Atlantic salmon has to our knowledge not been investigated. Cross adaptation experiments have shown that bile acids and prostaglandin F_2α_ have different olfactory receptors in lake charr (*Salvelinus namaycush*) (Zhang and Hara 2009) and in brown trout amino acids and prostaglandin F_2α_ have different receptors (Laberge and Hara 2004). Not all salmonids detect prostaglandin F_2α_ and other prostaglandins (Hara and Zhang 1998; Laberge and Hara 2003).

There is evidence that both vibrational and visual signals are important for salmonid male spawning behavior (Moore and Waring 1999; Newcombe and Hartman 1980; Satou et al. 1987). Moore and Waring (1999) observed that the sound of a red cutting female increased the amount of expressible milt in Atlantic salmon parr as much as after exposure to PGF_2α_. It has been suggested that olfactory signals can be important to maintain male arousal and to provide information about females’ readiness to spawn (Olsén and Liley 1993; Liley et al. 1993). In a study with kokanee salmon (*Oncorhynchus nerka*) in net pens in their spawning creek, plasma gonadotropin levels and volumes of expressible milt were higher in dominant kokanee males attending females compared with subordinate males not attending females (Liley et al. 1993). Anosmic males were less vigorous and persistent in their courtship and showed marked reduction in milt volumes. They still were as aggressive as the sham-operated controls during interaction with other males. In the present study with brown trout parr, the males were probably not completely anosmic after IPBC treatment and, in combination with recovery of the olfactory sense, the female priming pheromone PGF_2α_ gave increased milt volumes. Our results do not rule out that IPBC contamination of natural waters can have acute or chronic effects on behaviors mediated by the olfactory sense. The results show that a 96-h exposure to IPBC followed by behavior tests in clean water did not result in any significant negative effects on the variables recorded. An acute supply of IPBC to the experimental spawning tank (cf. Tierney et al. 2006a, b) could have affected the fish, but then, not only the precocious males would have been exposed. Furthermore, it was not possible to add IPBC directly in to the 15,000-l stream tank as the water drained into the river and could have affected salmon and brown trout present downstream.

In conclusion, pre-exposure to the fungicide IPBC (3-iodo-2-propynyl butyl carbamate) 15 μg l^−1^ did not reveal any significant effects on milt volume nor on time with nest-digging females in precocious brown trout male parr. IPBC has in previous studies affected olfactory responses to amino acids (EOG) and behavioral responses to l-histidine and skin extracts in juveniles of rainbow trout and coho salmon. In these tests, IPBC was added directly to the trough, and the behavior tests were run directly after or during exposure. Tests with another carbamate pesticide, carbofuran, have shown reduced EOG responses and olfactory sensitivity to prostaglandins and negative effects on their olfactory priming effects in mature male Atlantic salmon parr after 4-day exposure. Our prediction was that exposure to IPBC should have resulted in reduced courting behavior and milt volumes in precocious brown trout parr, but we could not demonstrate any negative effects.
